# Strong Cavity-Optomechanical Transduction of Nanopillar Motion

**DOI:** 10.1021/acsnano.4c09014

**Published:** 2024-08-21

**Authors:** Juliana Jaramillo-Fernandez, Martin Poblet, David Alonso-Tomás, Christian Vinther Bertelsen, Elena López-Aymerich, Daniel Arenas-Ortega, Winnie Edith Svendsen, Néstor Capuj, Albert Romano-Rodríguez, Daniel Navarro-Urrios

**Affiliations:** † Departament d’Enginyeria Electrònica i Biomèdica, Universitat de Barcelona, 08028 Barcelona, Spain; ‡ Institute of Nanoscience and Nanotechnology (IN2UB), Universitat de Barcelona, 08028 Barcelona, Spain; § DTU Bioengineering, Danmarks Tekniske Universitet (DTU), 2800 Kgs. Lyngby, Denmark; ∥ DTU Nanolab, Danmarks Tekniske Universitet (DTU), 2800 Kgs. Lyngby, Denmark; ⊥ Depto. Física, Universidad de La Laguna, 38200 San Cristóbal de La Laguna, Spain; # Instituto Universitario de Materiales y Nanotecnología, Universidad de La Laguna, 38071 Santa Cruz de Tenerife, Spain

**Keywords:** nanopillars, nanowires, mechanical resonators, force sensors, cavity optomechanics

## Abstract

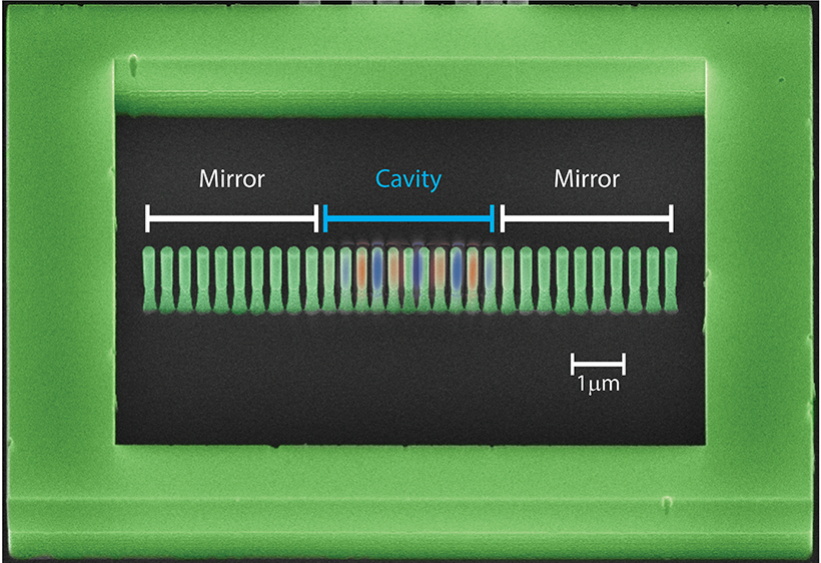

Nanomechanical resonators can serve as ultrasensitive, miniaturized force probes. While vertical structures such as nanopillars are ideal for this purpose, transducing their motion is challenging. Pillar-based photonic crystals (PhCs) offer a potential solution by integrating optical transduction within the pillars. However, achieving high-quality PhCs is hindered by inefficient vertical light confinement. Here, we present a full-silicon photonic crystal cavity based on nanopillars as a platform for applications in force sensing and biosensing areas. Its unit cell consists of a silicon pillar with a larger diameter at its top portion than at the bottom, which allows vertical light confinement and an energy band gap in the near-infrared range for transverse-magnetic polarization. We experimentally demonstrate optical cavities with *Q* factors exceeding 10^3^, constructed by inserting a defect within a periodic arrangement of this type of pillars. Each nanopillar naturally behaves as a nanomechanical cantilever, making the fabricated geometries excellent optomechanical (OM) photonic crystal cavities in which the mechanical motion of each nanopillar composing the cavity can be optically transduced. These geometries display enhanced mechanical properties, cost-effectiveness, integration possibilities, and scalability. They also present an alternative in front of the widely used suspended Si beam OM cavities made on silicon-on-insulator substrates.

Nanopillar mechanical resonators made from semiconductor materials have attracted significant attention due to their potential as extremely sensitive force sensors.^1−4^ These resonators, also referred to in the literature as vertical nanowires, possess exceptional mechanical properties, including very low mass, single-crystal quality, and controllable dimensions and geometry.^5^ Their high aspect ratio results in flexural mechanical modes that are extremely sensitive to external perturbations of different nature.^4^ However, transducing their motion presents a particular challenge since, to achieve their ultimate force sensitivity, the detection scheme must be able to measure the thermal motion.^4^ Unlike horizontally oriented nanoelectromechanical systems, which typically employ electrodes placed directly on top of the resonator,^6,7^ nanopillar motion requires high-resolution, complex optical imaging techniques for accurate characterization.^8−12^

Our approach to address this challenge relies on the construction of a one-dimensional photonic crystal (1D-PhC)^13^ with a linear array of nanopillars, thereby enabling an integrated optomechanical (OM) transduction scheme. Properly designed 1D-PhCs can exhibit a photonic band gap in the near-infrared telecom windows along their periodic direction, while at the same time confining light in the lateral and vertical dimensions through refractive index guiding.^13^ Effective light confinement in 1D-PhCs is typically achieved by introducing different dielectric materials to create a sufficiently large refractive index contrast between the 1D-PhC material and the surrounding media. However, since the initial proposals of 1D-PhCs,^13^ the exploration of nanopillar-based 1D-PhCs has been significantly limited compared to the nanobeam with hole counterparts.^14−18^ This limitation is largely due to the inherent challenges of achieving a sufficiently large refractive index contrast in the vertical dimension to prevent light leakage and the difficulty in fabricating vertical nanopillars that do not naturally bend. In this regard, PhC nanobeams have the advantage of being fabricated as free-standing structures surrounded by air, whereas nanopillars require anchoring to a substrate. For these reasons, there have been only a few experimental demonstrations of two-dimensional PhCs based on pillars^19−21^ and no experimental demonstrations of a 1D-PhC. Thus far, only numerical studies are documented in the literature that concern 1D-PhCs based on nanopillars,^22^ also known as rods in the photonic crystal research field.

In this article, we demonstrate a PhC geometrical configuration based on nanopillars that overcomes previous challenges and that can be monolithically fabricated on a single material wafer, e.g., monocrystalline silicon (Si) as in the present work. Indeed, we have successfully fabricated optical cavities by inserting an adiabatic defect within 1D-PhCs made of Si pillars which display experimental optical quality factors exceeding 10^3^ at wavelengths around 1.5 μm. Moreover, since the pillars constituting the cavity region are at the same time a collection of mechanical resonators, we experimentally demonstrate that the fabricated 1D-PhC pillar cavities are also excellent OM photonic crystal cavities that allow the efficient transduction of thermal motion associated with flexural modes up to the fourth order of every pillar composing the cavity. These geometries integrate the mechanical properties of semiconductor nanopillars within a high-quality photonic crystal cavity, offering a configuration for highly sensitive force sensors based on an OM transduction mechanism, which differs from purely mechanical nanowire cantilevers and PhC nanobeams.^23,24^

## Results and Discussion

**Figure 1:**
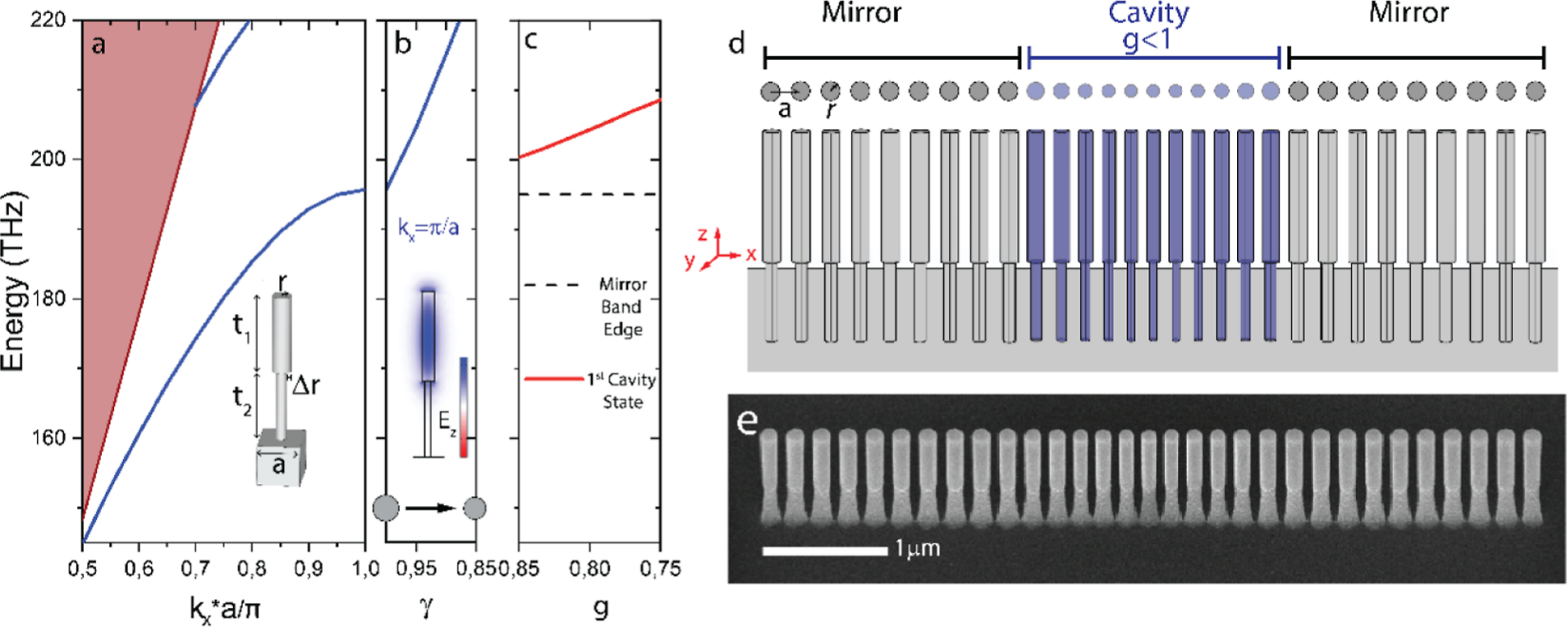
Photonic properties of a 1D-PhC cavity composed of a linear array of nanopillars. (a) Photonic dispersion relation showing the TM-like polarized optical modes (blue lines) of an idealized mirror unit cell (depicted in the inset). The shaded region is the light cone. The 1D-PhC has a band gap for TM-like modes, centered around 200 THz. The unit cell has a lattice constant *a* = 350 nm, pillar radius *r* = 105 nm, and Δ*r* = 50 nm, and the top and bottom portions of the pillars have heights *t*_1_ = *t*_2_ = 1500 nm. (b) Dependence of the *X*-point band edge energy on the reduction factor γ. The spatial distribution of the electric field along the vertical direction of the pillar (E_*z*_) is also illustrated for the state at the band edge. (c) Dependence of the energy of the optical cavity state on the depth of the defect region parameterized in terms of the reduction factor *g*, which determines the radius and pitch of the pillars at the center of the cavity. The optical cavity state is situated above the band edge (illustrated with a dashed line). (d) Schematic illustration of the geometrical parameters of the photonic crystal waveguide cavity as seen from the top and from the side. (e) Scanning electron microscope tilted view (30°) of a representative fabricated 1D-PhC pillar cavity with scaling factor *g* = 0.75. Note that γ is a scaling factor of the whole geometry, while *g* is the defect depth, i.e., ratio of the dimensions of the central cell with respect to those of a mirror cell.

The unit cell of the 1D-PhC comprises monocrystalline silicon (Si) pillars with two vertical portions: a top Si portion with a height *t*_1_ resting on top of a lower Si portion, with a height *t*_2_. The radius of the top portion, denoted as *r*, is approximately Δ*r* = 50 nm larger than that of the bottom portion (refer to the inset of Figure 1a). The substrate is also composed of silicon, although employing materials like silicon oxide (SiO_2_), with lower refractive index, would have negligible impact on the photonic behavior for the geometry employed here. The unit cell is repeated with a pitch (a) along the propagation direction (*x* axis). The photonic dispersion relation associated with the previous geometry, which is shown in the main panel of Figure 1a for the transverse-magnetic (TM)-like polarized optical modes (blue lines), displays a band gap for light with TM-like polarization and does not support transverse-electric (TE)-like modes. By choosing the right geometrical parameters (*a* = 350 nm, *r* = 105 nm, and *t*_1_ = *t*_2_ = 1500 nm), the TM-like band gap can be positioned around 200 THz, with a gap-to-midgap ratio of 5.7%. The electric field distribution along the pillar axis for the lowest energy band at its band edge at *k*_*x*_ = π/*a* (refer to the inset of Figure 1b) is spatially localized in the top portion of the pillar, thus being isolated from the presence of the Si substrate. In the main panel of Figure 1b, we have plotted the energy of this optical mode as a function of a factor γ that rescales *r* and *a*, keeping the pillar height and Δ*r* unchanged. As expected, the band edge is pushed up in energy by decreasing γ, thus providing the needed intuition to create a defect within the 1D-PhC that would place a resonant optical mode within its photonic band gap. Importantly, for heights of the lower portion of the pillar above a minimum value of *t*_2_ ∼ 200 nm, no modification in the band diagram occurs if the rest of the geometrical parameters are unchanged.

The 1D-PhC pillar cavity is composed of two mirrors, each one of them consisting of 9 equivalent cells with the geometrical parameters associated with the band dispersion of Figure 1a. The defect region has been placed between the mirrors by inserting 10 or 11 central cells in which the pitch and the radius are progressively reduced from the outer cells in a quadratic way toward the center to a minimum value of *g* × *a* and *g* × *r*, respectively. It is noteworthy that the energy of the fundamental optical cavity state can be tuned by varying the defect depth described by the factor *g* (Figure 1d). A deeper defect (corresponding to a smaller scale factor *g*) results in higher energy of the optical cavity state. Here, we have also tested the possible influence of variations of *t*_1_ and *t*_2_ (see Supporting Information Section S3), concluding that, above *t*_2_ ∼ 200 nm, the optical quality factor (*Q* factor) improves with *t*_2_, while the resonance position remains unchanged. For *t*_2_ < 200 nm, radiative leaking toward the Si substrate prevents supporting any cavity mode. Importantly, for these latter geometrical parameters (*t*_2_ < 200 nm), the expected *Q* factor is limited to 10^2^ due to radiative leaking even by employing materials with low refractive index like SiO_2_ (see Supporting Information Section S3). This limitation justifies the absence in the literature of experimental realizations of pillar-based 1D-PhC geometries. On the other hand, for heights of the higher portion of the pillar above *t*_1_ ∼ 500 nm, the optical *Q* factor increases, while the energy of the mode decreases. For *t*_1_ < 500 nm, the mode is cutoff, i.e., the mode leaks into the surrounding materials and attenuates. An illustration of the top and side view of a representative 1D-PhC pillar cavity with *g* = 0.75, *a* = 350 nm, *r* = 105 nm, *t*_1_ = 1580 nm, and *t*_2_ = 850 nm is displayed in Figure 1d.

The 1D-PhC pillar structures were fabricated by electron beam (e-beam) lithography and reactive ion etching (RIE) on an n-type ⟨100⟩ Si substrate. The wafer was prepared for e-beam exposure by spin-coating a 180 nm layer of CSAR positive e-beam resist. A 20 nm aluminum layer was deposited using e-beam evaporation followed by a lift-off process to remove the resist, leaving the aluminum layer with the desired pattern as a mask for the subsequent dry etch. The RIE process was done in 2 steps to create the upper and lower portions of the nanopillar with different diameters. The first part of the etching process was performed with a simultaneous mix of etching gas and passivation gas. For the second part of the process, the etch was changed to a cyclic process alternating between etching and passivation. The height of the nanopillars can be adjusted by changing the etching time in the first step and the number of cycles in the second step. Finally, the remaining aluminum mask was removed using a developer. Figure 1e shows a scanning electron microscopy image of one of the resulting geometries. More details on the fabrication recipes can be found in Supporting Information Section S2.

In our experiments, the 1D-PhC pillar cavity was replicated across multiple chips, varying the defect depth by factors of 0.85, 0.8, and 0.75. Additionally, the entire 1D-PhC cavity was scaled by a factor γ ranging from 1 to 1.1 in steps of 0.02. This allowed the mapping of the energy of the optical cavity modes and the resulting variations in the mechanical response.

### Optical Properties of the 1D-PhC Pillar Cavity

**Figure 2:**
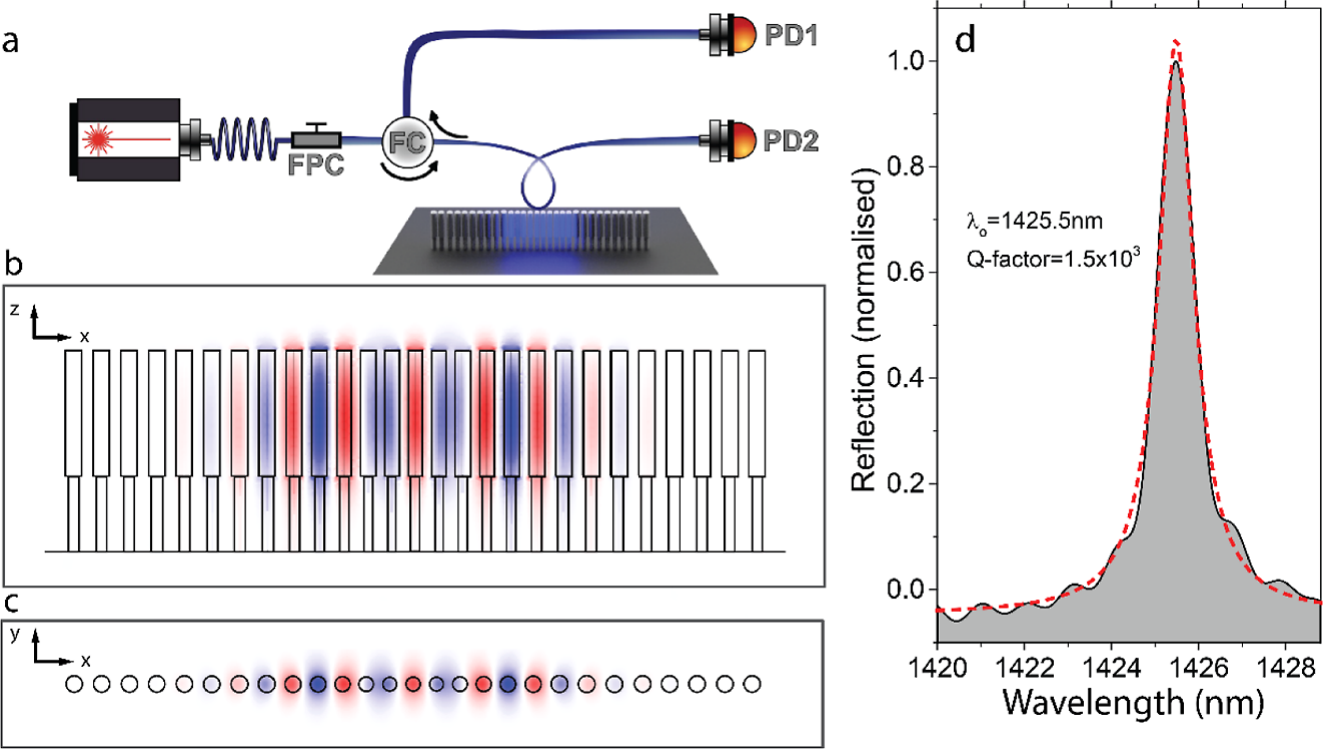
Optical properties of the 1D-PhC pillar cavity. (a) Schematic of the experimental setup. The fiber passes through a fiber polarization controller (FPC) and a fiber circulator, enabling reflection and transmission experiments, detecting with the photodetector (PD) 1 and 2, respectively. The fiber loop is positioned over the cavity region. Dimensions are not to scale. The diameter of the loop is about 50 μm, while the total length of the 1D-PhC pillar cavity is about 10 μm. (b,c) Finite-element-method (FEM) simulation of the electric field along the *z* direction (*E*_*z*_) of the TM fundamental optical cavity mode as seen from the side and from the top, respectively. (d) Experimental characteristic reflection spectrum of one of the fabricated geometries. The dashed line is a Lorentzian fit to the experimental data.

The optical properties of the samples were examined by evanescently coupling resonant laser light into the cavity. This was achieved using a fiber loop positioned above the cavity region, aiming to avoid physical contact with the pillars. An infrared driving laser with a tunable wavelength (1355–1640 nm) and power up to 20 mW passes through a polarization controller before reaching the fiber loop. All the measurements were performed in an antivibration cage at ambient conditions. A schematic of the experimental setup, which allows both reflection and transmission configurations, is presented in Figure 2a and described in detail in Supporting Information Section S1. The 1D-PhC pillar cavities are of the bidirectional type; i.e., light can decay in both forward and backward propagating modes of the fiber loop. Thus, it gives rise to an optical resonance that points upward in a reflection spectrum when the input light excites the cavity via the evanescent field. The optical reflection spectrum of a representative fabricated 1D-PhC pillar cavity with γ = 1.06 and *g* = 0.8 is shown in Figure 2d. By comparing the simulations and the experimental results, it is possible to identify that the observed resonance corresponds to the fundamental optical cavity mode of the set of cavity modes supported by this geometry, which would in principle lead to a quality factor exceeding 10^4^ (see Supporting Information Section S3). Figure 2b,c is, respectively, the top view and cross section of the spatial profile of the simulated fundamental TM optical cavity mode above the band edge of a 1D-PhC pillar cavity made with the idealized mirror cell as described in Figure 1c. For this cavity mode, the electric field is strongly localized in the cavity center and decays rapidly in the mirror regions.

In the experiment, the optical resonance wavelength is centered at around 1365.5 nm. The Lorentzian fit to the experimental data (refer to the dashed curve of Figure 2d) allows estimating that the fabricated structure owns an overall optical quality factor of *Q* = 1.5 × 10^3^. This value is very close to the intrinsic one considering that the coupled power fraction is rather low (<10%), resulting from a significant effective index mismatch between the propagating mode of the tapered fiber (*n*_eff_ ∼ 1.4) and the cavity state (*n*_eff_ ∼ 2.1). Intrinsic losses are, thus, dominating the overall *Q* factor of the cavity; i.e., extrinsic losses due to the evanescent light coupling to the fiber can be neglected. The intrinsic losses of the fabricated 1D-PhC pillar cavities probably stem from scattering losses at the surface of the pillars, mainly localized at the region where the supported mode overlaps with the undercut region, i.e., where the roughness of the pillar surface significantly increases. It is also likely that single-photon absorption due to intragap electronic states related to dangling bonds at the pillar surface plays a significant role in limiting the optical *Q* factor. Possible strategies to improve these values rely on the passivation and smoothing of the pillar surface, which may reduce at once light absorption and surface scattering.

### Mechanical Response of the 1D-PhC Pillar Cavity

**Figure 3:**
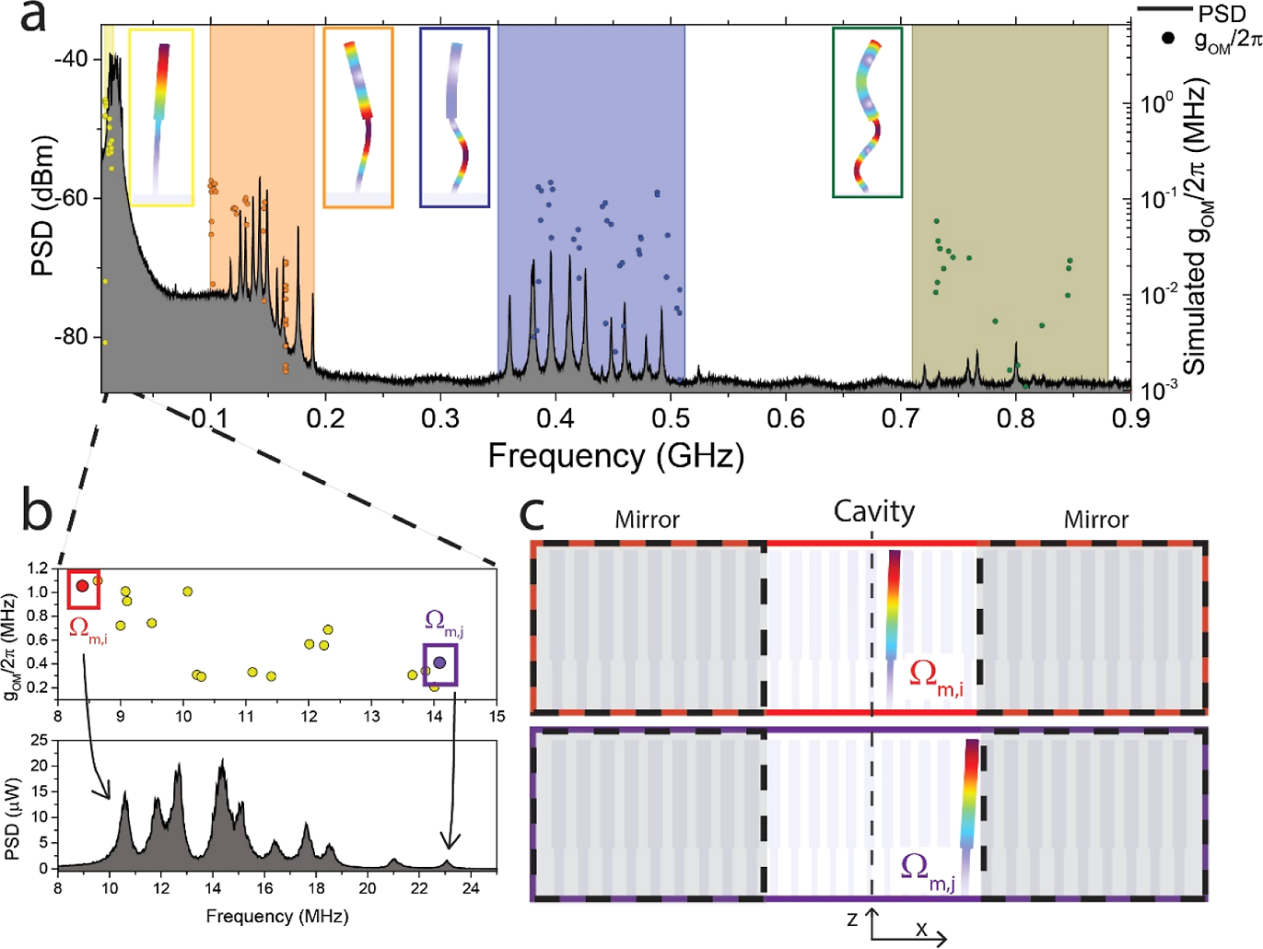
Mechanical spectrum of the 1D-PhC pillar cavity measured through OM transduction. (a) RF spectrum, acquired by OM measurement, evidencing the mechanical modes of vibration transduced by the 1D-PhC pillar cavity. Four different families of cantilever-like modes, highlighted by color boxes, can be identified from FEM simulations for the fundamental and up to the fourth harmonic. The related displacement field profile for each family is shown in the insets. The right axis and the scattered dots correspond to the vacuum OM coupling rate (*g*_OM_/2π) calculations for a representative 1D-PhC pillar cavity of γ = 1 and defect depth *g* = 0.75 with dimensions corresponding to the fabricated and measured geometry. (b) Zoom of the fundamental family region. The experimental power spectral density (PSD) is plotted in the linear scale in this case. Two mechanical modes placed at the extremes of the analyzed spectral band are highlighted with colored squares, whose deformation profile is depicted in panel (c) using the same color code.

When light is coupled to an optical mode such as that reported in Figure 2d, the thermally activated mechanical oscillation modes of the 1D-PhC pillar cavity can be optically transduced. This measurement relies on the dependence of the spectral position of the optical resonance with a pillar deformation, namely, the OM coupling, which leads to a modulation of the transmitted or reflected light that can be detected with large bandwidth near-IR photodetectors and processed with a spectrum analyzer.^25^ The radiofrequency (RF) spectrum evidencing the mechanical response of a representative 1D-PhC pillar cavity of γ = 1 and defect depth *g* = 0.75 is reported in Figure 3a. This data is superimposed to the vacuum OM coupling strength (*g*_OM_/2π) simulated with a FEM solver plotted on the right axis.

The PSD spectrum displays multiple mechanical modes appearing as narrow Lorentzian peaks. Within the 1D-PhC pillar cavity, each individual pillar serves as a mechanical resonator anchored at one end (the substrate), vibrating at its own mechanical resonance frequency (Ω_m,i,_ where subindex i indicates a specific pillar of the cavity region). The mechanical signal exhibits a significant complexity, showcasing at least four families of cantilever-like modes defined over specific spectral bands. Due to variations in the radii of the pillars forming the cavity, multiple peaks are observed within the spectral band covered by each family.

The related displacement field profile for the four families of cantilever-like mechanical modes is shown in the insets of Figure 3a. The motion associated with these modes and the corresponding displacement field profiles have been identified through FEM simulations. These families extend from the fundamental mode family, occurring at the lowest frequencies (<50 MHz), up to the fourth harmonic mode family at higher frequencies (<900 MHz). The computed values of *g*_OM_/2π are dominated by a moving interface contribution^26^ despite the photoelastic one (see Supporting Information Section S4).^27^ Notably, the overall values exceed 1 MHz for some of the mechanical modes within the first family of modes.

Even though the FEM simulations were performed using an idealized pillar profile (Figure 1d) that slightly deviates from the fabricated one mostly on the undercut region, it is evident from Figure 3a that the computed frequencies for the four distinct mode families are in reasonably good agreement with the experimental observations. Interestingly, the PSD signal spans over 5 orders of magnitude with the larger amplitudes belonging to the family of the fundamental modes and the weaker ones, corresponding to the fourth family of modes. This fact can be explained mainly by the relative magnitudes of the g_OM_ values calculated for each family. Indeed, the PSD signal is proportional to *g*_OM_^2^*n*_th_,^28^ where n_th_ is the frequency-dependent average mechanical occupation number. In the high temperature limit, *n*_th_ ≈ *k*_B_*T*_bath_/ℏΩ_m,i_, where *T*_bath_ is the ambient temperature and *k*_B_ and ℏ the Boltzmann and Planck constants, respectively.^25,28^

Within each family, the pillars with smaller radii have smaller Ω_m,i_ compared to pillars with larger radii, i.e., the mechanical modes within the cavity region have Ω_m,i_ of decreasing values as the pillars are localized closer to the center of the 1D-PhC pillar cavity. Figure 3b illustrates this for the case of the fundamental family of mechanical modes by zooming in on the low-frequency region of Figure 3a. The experimental signal (bottom panel of Figure 3b) thus consists of a set of resonances, each corresponding to one pillar, with the frequency increasing for pillars located farther from the center. To support the previous statement, in Figure 3c, we show the deformation profile of modes situated at the two extremes of the fundamental family spectral band according to FEM simulations. The spatial distribution of the moving interface contribution to the *g*_OM_ values is localized at the specific oscillating pillar, as expected (see Supporting Information Section S4). In force sensing or biosensing applications, tracking the spectral shift of a mechanical RF peak could be directly related to perturbations at the location of the pillar providing that signal. This would enable extraction of spatial maps of the perturbations, with a resolution determined by the spacing between adjacent pillars. These features are in sharp contrast to that displayed by suspended 1D-PhC nanobeam cavities,^18,27,29^ where the supported mechanical modes involve a collective oscillation of most of the cells composing the cavity.

The mechanical quality factors of the observed modes are on the order of 1 × 10^1^ for the first family of modes and 1 × 10^2^ for the rest of the families. Given the frequency range of the modes and the humidity of our lab (between 45 and 50%), mechanical losses are likely dominated by viscoelastic losses due to interaction with the surrounding medium.^30^

**Figure 4:**
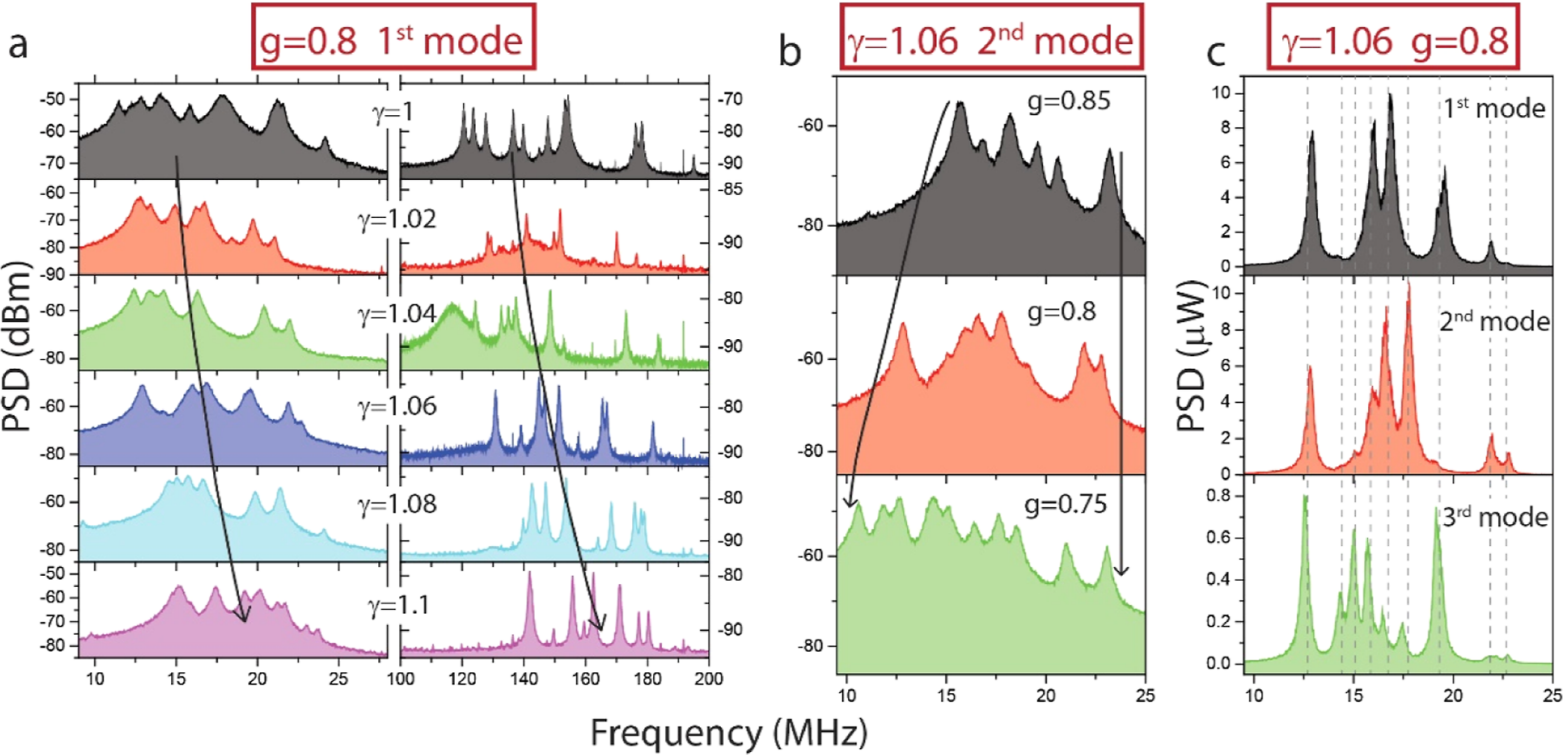
Mechanical spectrum dependence of the geometrical parameters. (a) RF spectrum for different values of the scaling factor γ for the fundamental and second-order family of mechanical modes (left and right, respectively). The defect depth is *g* = 0.8 and the optical mode under test is the fundamental one. Black arrows indicate the general tendency of the set of observed peaks. (b) RF spectrum for different values of the defect depth g for the fundamental family of mechanical modes. The scaling factor is γ = 1.06 and the optical mode under test is the second-order one. Black arrows indicate the general tendency at the extremes of the spectrum. (c) RF spectrum for a fixed geometry and different optical modes under test. The scaling factor is γ = 1.06 and the defect depth is *g* = 0.8. Black dashed lines indicate the spectral position of the mechanical modes. PSD is reported in the linear scale in this case.

To provide further insights into the effect of the geometrical parameters of the 1D-PhC pillar cavities on their mechanical properties, we have made a systematic study of the behavior of the mechanical spectra upon variation of the overall scaling factor γ and the depth of the cavity region g (see Figure 4a,b, respectively), keeping the other parameters fixed.

In the first case, varying the γ factor (see Figure 4a), we observe that every family of mechanical modes (here only the first two are shown) increases its average frequency by increasing γ, which is indicated with the black arrows. This is an expected result that is consistent with the general behavior of individual mechanical cantilevers, wherein a larger radius correlates with a higher oscillation frequency. It is worth noting that in this set of experiments, we are also fixing the order of the optical mode under test, which in Figure 4a is the fundamental one.

When g is adjusted while γ is kept constant (see Figure 4b), we notice that the higher-frequency side of the spectrum remains largely unchanged, whereas there is an expansion observed on the lower-frequency side as g decreases. This is coherent with the fact that deeper defects are produced by smaller pillar radii near the center of the cavity, resulting in lower natural frequencies in that region. Conversely, pillars located at the cavity periphery exhibit minimal variations in response to modifications of *g*. We focused the set of experiments performed in Figure 4b on the fundamental family of mechanical modes and used the second-order optical mode. The behavior of the mechanical frequencies with *g* is indicated with black arrows.

Finally, in Figure 4c, we have fixed the geometrical parameters of the 1D-PhC pillar cavity and explored the variations of the mechanical spectrum, focused on the fundamental family, as the different supported optical cavity modes are excited. In this case, given that we are dealing with the same geometry, we observe that the set of transduced mechanical modes remains consistent across the panels, as denoted by the dashed vertical lines indicating their spectral position. However, the relative signal strength associated with each mechanical peak varies among the figure panels. This variability directly stems from the different electromagnetic field spatial distribution along the 1D-PhC pillar cavity for the distinct supported optical modes, an observation that is also reinforced with FEM simulations (Supporting Information Section S4).

### Sensing External Forces: Physically Perturbing the 1D-PhC Pillar Cavities with the Tapered Fiber

**Figure 5:**
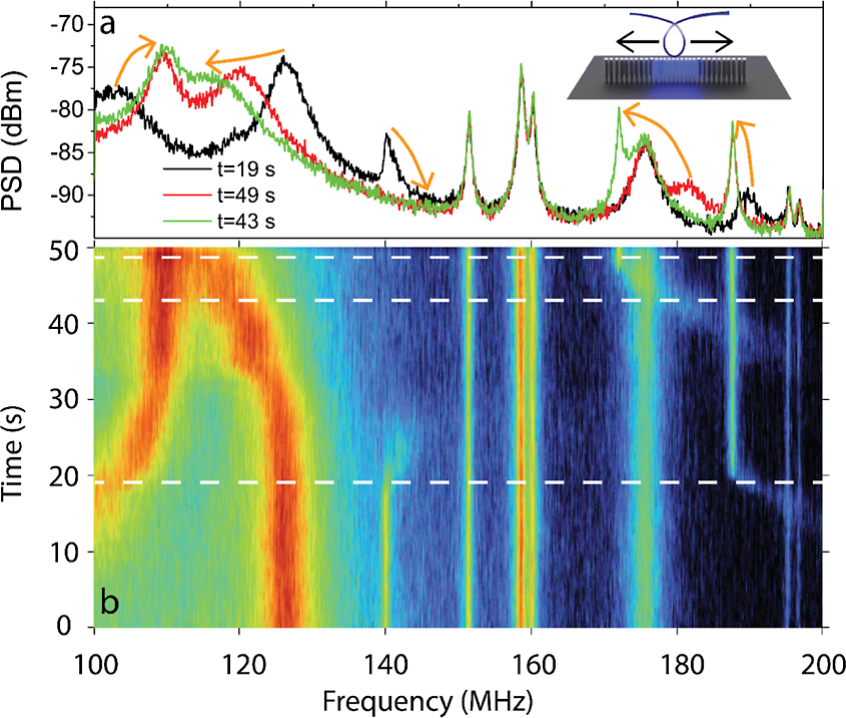
Evolution of the mechanical spectrum with the positioning of the tapered fiber on top of the 1D-PhC pillar cavity while in physical contact. (a) RF spectrum for three different positions of the fiber, with arrows indicating the evolution of the peaks across the measurements. The inset shows a sketch of the relative positioning between the fiber and the geometry. (b) Temporal evolution of the RF spectrum as the fiber is manually dragged back and forth over the tops of the pillars. The dashed lines indicate the moments at which the RF spectra displayed in panel (a) were taken.

To illustrate the sensitivity of the 1D-PhC pillar cavities to physical perturbations, we brought the tapered fiber used for optical probing into contact with the pillars. We recorded the evolution of the RF spectrum, focusing on the spectral band covered by the second family of mechanical modes, while manually dragging the fiber back and forth along the *x* direction over the tops of the pillars (see Figure 5). Interestingly, several RF peaks were perturbed and significantly shifted in spectral position during the measurement. We attribute the observed line width broadening of these perturbed RF peaks to the damping of mechanical modes caused by physical contact with the fiber. Supporting our interpretation, some of the broad peaks around 180 MHz shift as the fiber is dragged and then suddenly become narrow and stable. We associate this behavior with the moment the fiber stops touching the specific pillars generating those RF peaks, thus recovering their free oscillation frequency and quality factor. Additionally, we observe that contact with the fiber increases the oscillation frequency of the pillars, which is compatible with an effective increase in their elastic constant.^4^ Finally, it is worth mentioning that the two RF peaks appearing below 140 MHz are associated with modes that, in their free oscillation configuration, belong to the first family. In this case, the contact with the fiber acts more like a clamping point, causing their frequency to increase by several tens of MHz. Figure 5a shows three RF spectra recorded for different positions of the fiber over the 1D-PhC pillar cavity, corresponding to the moments highlighted in Figure 5b with white dashed lines. The quantification of the interaction between the fiber and the pillars leading to the observations of Figure 5 is beyond the scope of this manuscript. Further insight into the sensitivity of the 1D-PhCs to force derivatives against nanopillar displacement using FEM simulations is provided in Supporting Information Section S5.

## Conclusions

In this work, we aimed at addressing the challenge of transducing the motion of nanopillar mechanical resonators, which are known for their exceptional mechanical properties and potential as highly sensitive force sensors. Our proposed solution is based on the construction of photonic crystal waveguide (1D-PhC) cavities composed of nanopillars, enabling integrated OM transduction. By carefully engineering the dimensions of the silicon pillars, we achieved vertical light confinement and established an energy band gap in the near-infrared spectrum for TM polarization. Through experimental demonstrations, we established optical cavities with quality factors exceeding 10^3^ by incorporating defects within the periodic one-dimensional arrays of pillars. We acknowledge challenges associated with optical scattering losses likely associated with the surface roughness of the undercut portion of the pillars and single-photon absorption from surface electronic states. However, our understanding of the mechanical and optical properties of these 1D-PhC pillar cavities can be applied to mitigate this issue and further optimize their optical performance.

Our approach leverages the dual functionality of nanopillars, which serve both as mechanical resonators and as photonic crystal cavities. This enables the efficient optical transduction of mechanical motion, with our structures exhibiting OM coupling rates large enough to enable the detection of thermal motion up to the fourth order of cantilever-like modes. These combined features could, for instance, enable noninvasive, label-free means of sensing variations of the pillar mechanical properties upon forces exerted by, for instance, biological specimens deposited on the surface of the PhC pillar cavities. Furthermore, since there exists a direct correlation between the oscillation frequency of a pillar and its spatial position within the cavity, achieving spatial resolutions on the order of the pitch, i.e., a few hundred nanometers, would be achievable.

Our design offers several advantages, including enhanced mechanical properties, cost-effectiveness, integration possibilities, and scalability. Future developments, such as the integration of an on-chip waveguide, will further enhance the practicality of our setup by eliminating the need for a tapered fiber.

The integrated transduction mechanism of the mechanical motion sets our 1D-PhC pillar cavities apart from previous efforts performed on nanowire sensors, simplifying the measuring process. Notably, our structures provide an alternative approach to conventional suspended Si beam OM cavities fabricated on silicon-on-insulator substrates.

In summary, this work advances the development of practical photonic devices based on PhCs composed of nanopillars with enhanced functionalities with potential applications in force sensing, biosensing, and related fields.

## Methods

### Experimental Setup Details

The experiments were made in a standard setup to characterize optical and mechanical properties of OM cavities illustrated in Figure 2a of the main text. To cover the wavelength range from 1355 nm up to 1640 nm, two lasers were used: a Santec TSL-570 for the range between 1355 and 1490 nm and a Yenista TUNICS T100S for the range from 1440 nm up to 1640 nm. The tapered silica fiber is connected to these lasers, and it first passes through an FPC and then a circulator. The FPC enables us to adjust the polarization of the input light to optimize coupling efficiency and minimize polarization-dependent losses. The circulator enables light to travel through each port in only one direction, and it is used to separate the transmitted and reflected light. Each branch is connected to a photodetector, which measures the power of the transmitted/reflected light at each wavelength.

To position the fiber loop on the cavity region, we use a 50× microscope objective and a CCD camera to capture zenithal images. The positioning is manually adjusted with a submicrometer precision positioning system.

To check for the presence of a RF modulation of the optical signal, we connect the output of the photodetectors to the 50 Ω input impedance of a spectrum analyzer with a bandwidth of 13.5 GHz. All the measurements were performed in an antivibration cage at ambient conditions.

### Fabrication Details

The 1D-PhC waveguide pillar structures were fabricated by e-beam lithography and RIE on an n-type ⟨100⟩ silicon substrate from Siegert Wafer (Germany) with a specified resistivity of 1–20 Ω cm (refer to Figure S1 of the Supporting Information file).

The wafer was prepared for e-beam exposure by spin-coating a 180 nm layer of a CSAR positive e-beam resist (AR-P 6200, Allresist GmbH, Germany). The desired pattern was exposed using a JEOL JBX-9500FS e-beam lithography system with a dose of 300 μC/cm^2^ and subsequently developed using developer AR 600-546 (Allresist GmbH, Germany) for 60 s. A 20 nm aluminum layer was deposited using e-beam evaporation followed by a lift-off process (Microposit Remover 1165) to remove the remaining CSAR resist, leaving the aluminum layer with the desired pattern as a mask for the subsequent dry etch.

The RIE process was done in 2 steps to create the upper and lower sections of the nanopillar with different diameters. The first part of the etching process was done with a simultaneous mix of etching gas (SF6 at 44 sccm) and passivation gas (C4F8 at 77 sccm) for 4 min with a coil power of 1000 W and a platen power of 20 W. For the second part of the process, the etch was changed to a cyclic process alternating between etching with SF6 and passivation with C4F8 for 25 cycles. The height of the nanopillars can be adjusted by changing the etching time in the first step and the number of cycles in the second step. Finally, the remaining aluminum mask was removed using a TMAH-based developer (AZ 726 MIF, MicroChemicals GmbH, Germany).

## Supporting Information Available

Figures including a sketch of the required fabrication steps to produce the 1D-PhCs, optical modes of 1D-PhC pillar cavities, evolution of the optical Q factor with the height of the bottom part of the pillar, evolution of the optical Q factor and spectral position of the optical mode with the height of the upper part of the pillar, OM coupling between the fundamental optical mode and mechanical modes of the pillars belonging to the cavity region, FEM simulations, and vacuum OM coupling calculations; design and optimization of the 1D-PhC geometries in terms of the optical, mechanical, and OM parameters using FEM simulations; and simulated response of the mechanical mode spectral position upon the application of external forces (PDF)
