# Supplementary material for: Strong Cavity-Optomechanical Transduction of Nanopillar Motion
Source: ACS Nano. 2024 Aug 21;18(35):24550–7. doi: 10.1021/acsnano.4c09014 (PMC11375771; doi:10.1021/acsnano.4c09014)
Supplement: Supplementary file 1 — nn4c09014_si_001.pdf [file nn4c09014_si_001.pdf]

Supplementary Information: Strong cavity-optomechanical transduction of nanopillar motion

Juliana Jaramillo-Fernandez[a,b,†], Martin Poblet[a,b], David Alonso-Tomás[a,b], Christian Vinther Bertelsen[c], Elena López-Aymerich[d], Daniel Arenas-Ortega[a,b], Winnie Edith Svendsen[c], Néstor Capuj[e,f], Albert Romano-Rodríguez[a,b,*], Daniel Navarro-Urrios[a,b,*]

[a] Departament d'Enginyeria Electrònica i Biomèdica, Universitat de Barcelona, 08028, Barcelona, Spain

[b] Institute of Nanoscience and Nanotechnology (IN2UB), Universitat de Barcelona, 08028, Barcelona, Spain

[c] DTU Bioengineering, Danmarks Tekniske Universitet (DTU), 2800 Kgs. Lyngby, Denmark

[d] DTU Nanolab, Danmarks Tekniske Universitet (DTU), 2800 Kgs. Lyngby, Denmark

[e] Depto. Física, Universidad de La Laguna, 38200 San Cristóbal de La Laguna, Spain

[f] Instituto Universitario de Materiales y Nanotecnología, Universidad de La Laguna, 38071 Santa Cruz de Tenerife, Spain

[†] Present address: Departament de Màquines i Motors Tèrmics, Universitat Politècnica de Catalunya, 08028, Barcelona, Spain

*Corresponding authors: albert.romano@ub.edu, dnavarro@ub.edu

This supplementary information file first includes a figure (Figure S1) sketching the required fabrication steps to produce the 1D-PhCs. It dedicates two sections to the design and optimization of the 1D-PhC geometries in terms of the optical, mechanical and optomechanical parameters using Finite-Element-Method simulations (Sections S2 and S3). The final section (Section S4) is dedicated to the simulated response of the mechanical mode spectral position upon the application of external forces. 8 pages, 4 sections, 7 figures.

S1. Fabrication steps

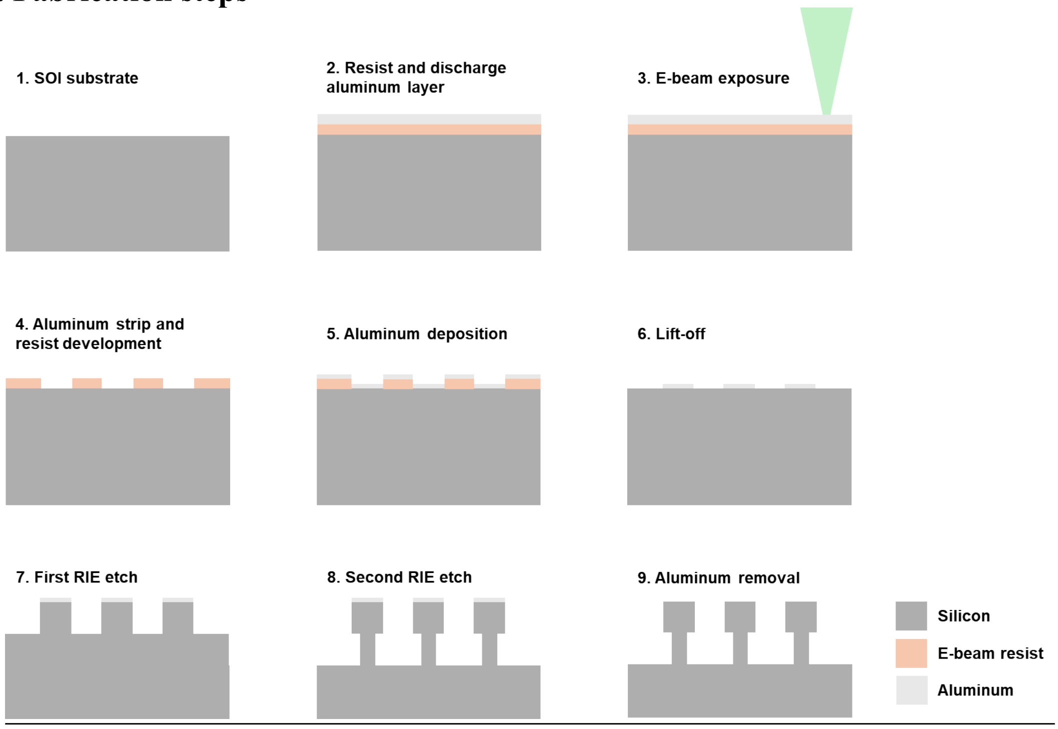

Figure S1. **Sketch of the fabrication steps required to produce the 1D-PhC pillar structures.**

S2. Optimization of the optical quality factor by design

In this section we discuss on the geometrical requirements to generate a high quality-factor optical state within a 1D-PhC made of silicon pillars. We use the optical quality-factor as a figure of merit for the optical cavities, which has been evaluated for the fundamental optical mode by calculating the ratio between the imaginary and the real parts of the optical eigenfrequencies.

The cavity region has been created as described in the main text. It consists of two mirror regions, each one of them constructed by a repetition of the same unitary cell about 9 times. Within them, we have introduced 11-12 cells with a gradual decrease of the pitch and radius of the pillars towards the centre. With this geometrical configuration of the optical cavity, the simplest cross section that can be simulated is that with the pillars surrounded by air, which is the case illustrated in Figure S2a. With this cross section it is possible to create cavity modes with radiative optical quality factors exceeding 10^5 at about 200 THz for pillar heights of t_l=1500nm. If the lower substrate is replaced by a solid one, such as SiO_2 as illustrated in Figure S2b, there appears a significant light leakage towards the substrate, limiting the quality factors of the cavity mode to below 10^3. We believe that this is the main reason explaining why there are no previous experimental realisations of 1D-PhC made of silicon pillars.

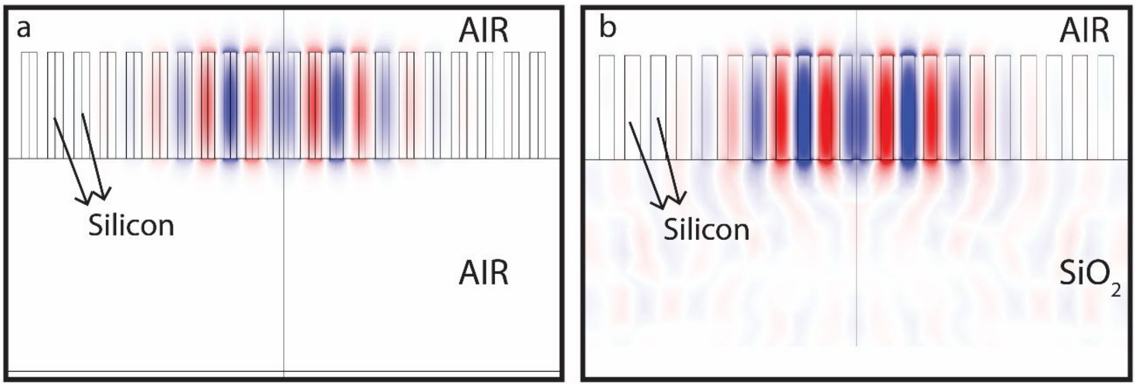

Figure S2. Optical modes of 1D-PhC pillar cavities. Finite-Element-Method simulation of the electric field along the z direction (E_z) of the TM fundamental optical cavity mode as seen from the side. a) The pillar array is surrounded by air. b) The pillar array leans on top of a SiO_2 substrate.

The previous results indicate that a geometrical innovation such as the one proposed by us in the main text must be introduced to obtain a high-quality 1D-PhC made of silicon pillars deposited on a substrate.

In the following studies we have fixed the silicon pillar geometry to that consisting of two vertically stacked silicon pillars, i.e., a top pillar of radius r with a height t_1 resting upon another Si pillar of radius r-Δr with a height t_2. The radii and pitch values have been fixed values to r=105nm, Δr=50nm and pitch a=350nm.

Figure S3 reports the evolution of the Q-factor as a function of the height of the lower pillar t_2, while t_1 has been fixed to t_1=1500nm. The optical Q-factor increases dramatically by improving the isolation from the substrate, i.e., by increasing t_2, while the mode frequency does not significantly shift (not plotted). If t_2<200nm the light leakage towards the substrate dominates, forbidding any supported optical mode confined on the top part of the pillar. The height of the fabricated structures (t_2~850nm) would ensure radiative optical Q-factor on the order of $5x10^4$, which is more than an order of magnitude greater than what measured experimentally. Therefore, the observed optical losses, which lead to experimental Q-factors of 10^3, probably stem from surface scattering losses at the lower part of the pillars.

It is worth noticing that, for t_2>200nm, FEM simulations indicate that the differences between using a Silicon or a SiO_2 as substrate are not significant. There are still supported modes in the case of using SiO_2 as substrate if t_2<200nm, but the Q-factors lie below 10^3. For a value of t_2=0, the results are those of Figure S1b.

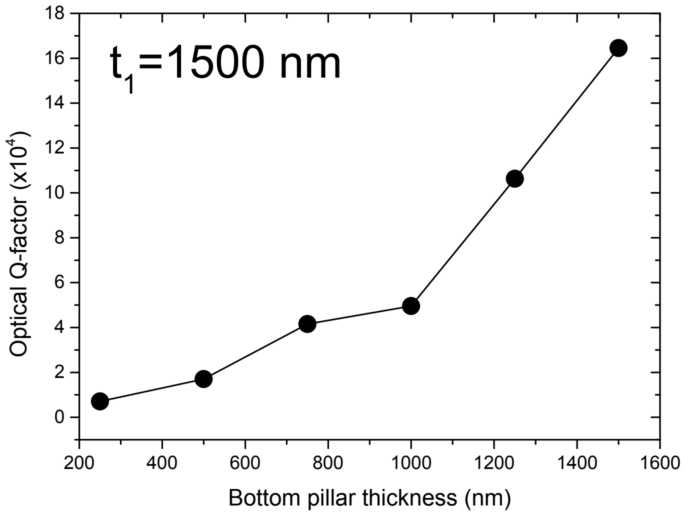

Figure S3. Evolution of the optical Q-factor with the height of the bottom part of the pillar. The height of the upper part of the pillar is fixed to t_1=1500nm

Finally, we have studied the effect of modifying the top pillar thickness t_1 while keeping t_2=1500nm. Figure S4 shows that, in this case, both the Q-factor (black curve, left axis) and the spectral position of the cavity mode (red curve, right axis) are affected, in a way that the former improves with t_1 while the latter shifts towards smaller frequencies. The cut-off for supporting an optical mode is found at t_1=500nm.

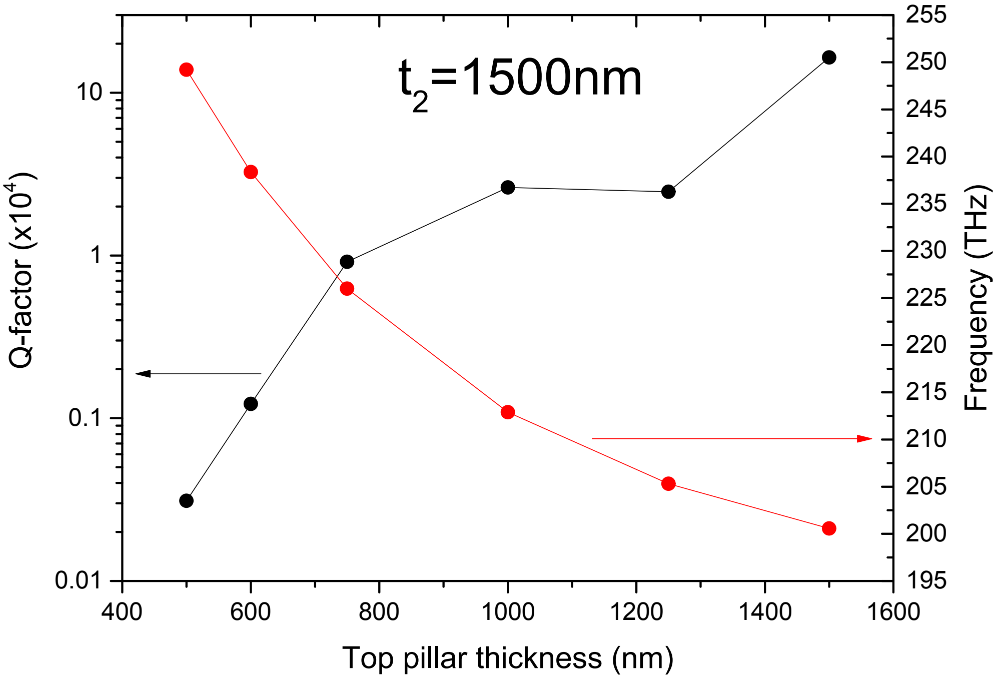

Figure S4. Evolution of the optical Q-factor (black curve, left axis) and spectral position of the optical mode (red curve, right axis) with the height of the upper part of the pillar. The height of the bottom part of the pillar is fixed at t_2=1500nm

S3. OM coupling calculations.

Single-particle optomechanical coupling rates (g_{OM}) between optical and mechanical modes are estimated by considering both photo-elastic (PE) and moving-interface (MI) effects[1,2]. The PE effect is a result of the acoustic strain within bulk silicon while the MI mechanism comes from the dielectric permittivity variation at the boundaries associated with the deformation.

The calculation of the MI coupling coefficient g_{MI} is performed using the integral given by Johnson et al.[1]:

$$g_{MI} = -\frac{\pi\lambda_r}{c}\frac{\oint (\boldsymbol{Q}\cdot\hat{\mathbf{n}})\left(\Delta\varepsilon \boldsymbol{E}_{\parallel}^2 - \Delta\varepsilon^{-1}\boldsymbol{D}_{\perp}^2\right)dS}{\int \boldsymbol{E}\cdot\boldsymbol{D}dV}\sqrt{\hbar/2m_{eff}\Omega_m}$$

(S1)

where **Q** is the normalized displacement (max{|**Q**|}=1), $\hat{\mathbf{n}}$ is the normal at the boundary (pointing outward), **E** is the electric field and **D** the electric displacement field. ε is the dielectric permittivity, $\Delta\varepsilon=\varepsilon_{silicon}-\varepsilon_{air}$, $\Delta\varepsilon^{-1}=\varepsilon^{-1}_{silicon}-\varepsilon^{-1}_{air}$. λ_r is the optical resonance wavelength, c is the speed of light in vacuum, $\hbar$ is the reduced Planck constant, m_{eff} is the effective mass of the mechanical mode and Ω_m is the mechanical mode eigenfrequency, so that $\sqrt{\hbar/2m_{eff}\Omega_m}$ is the zero-point motion of the resonator.

A similar result can be derived for the PE contribution[2]:

$$g_{PE} = -\frac{\pi\lambda_r}{c}\frac{\langle E|\delta\varepsilon|E\rangle}{\int \boldsymbol{E}\cdot\boldsymbol{D}dV}\sqrt{\hbar/2m_{eff}\Omega_m} \qquad \text{(S2)}$$

where $\delta\varepsilon_{ij}=\varepsilon_{air}\, n^4 p_{ijkl} S_{kl}$, being p_{ijkl} the PE tensor components, n the refractive index of silicon, and S_{kl} the strain tensor components.

The addition of both contributions results in the overall single-particle OM coupling rate. However, we have observed that the PE contribution to g_{OM} is negligible with respect to the MI one and thus we can assume that $g_{OM}\sim g_{MI}$.

To provide further insight on the optomechanical properties of the 1D-PhC cavity made of silicon pillars, in Figure S5 we represent the spatial contributions relevant for the calculation of g_{MI}, i.e., the integrand of Eq. S1. We have focused on the fundamental optical mode (refer to Figure S5a) and the mechanical modes highlighted in Figure 3 of the main text, which are associated to a pillar

close to the centre ($\Omega_{m,i}$=8.39MHz, refer to Figure S5b) and a pillar at the side of the cavity region ($\Omega_{m,j}$=14.09MHz, refer to Figure S5c), respectively. As expected, the contribution to g_{MI} is just confined to the oscillating pillar (refer to Figure S5d and Figure S5e), which implies that the resonant peaks that are measured in the RF spectra of the optical signal are associated to the mechanical oscillation of single pillars placed within the cavity region.

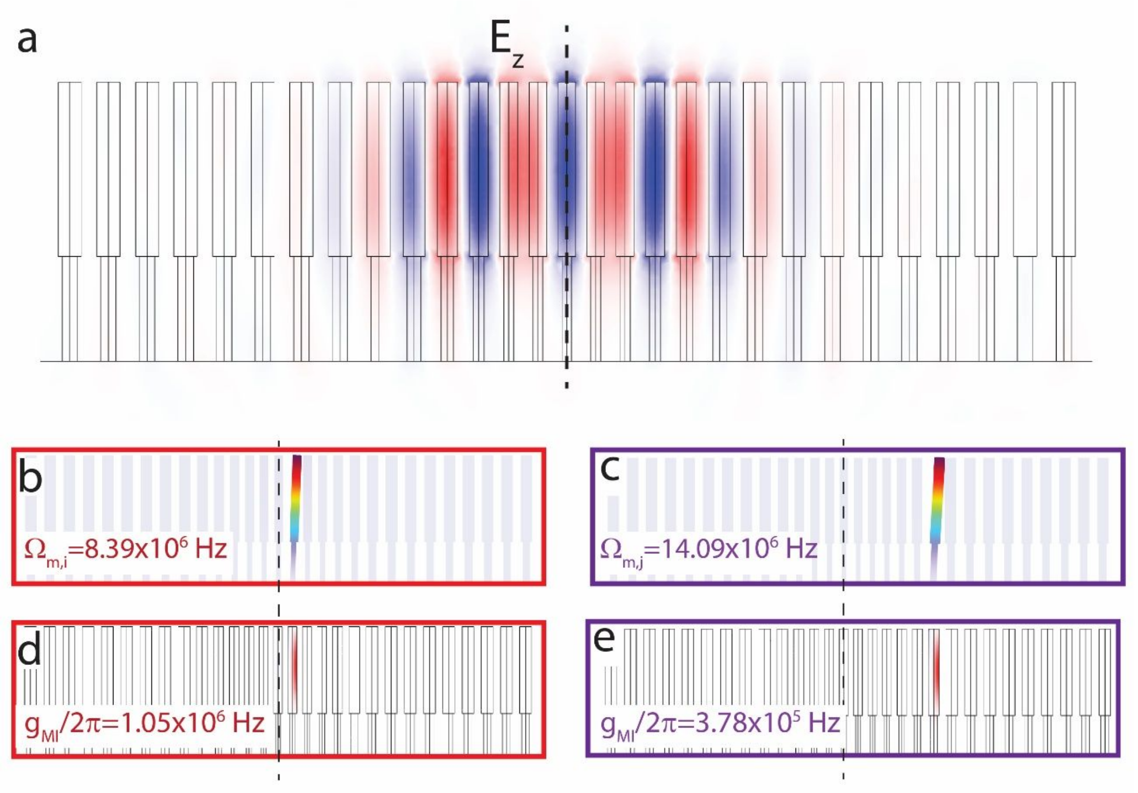

Figure S5. Optomechanical coupling between the fundamental optical mode and mechanical modes of the pillars belonging to the cavity region a) Finite-Element-Method simulation of the electric field along the z direction (E_z) of the TM fundamental optical cavity mode as seen from the side. b) and c) Normalized mechanical displacement field |Q| of mechanical modes at 8.39 MHz (panel b) and 14.09 MHz (panel c). d) and e) Normalized surface density of the integrand in Eq. S1, showing the contributions to g_{MI} associated to the modes of panels b and c, respectively.

It is worth noting that, outside the cavity region, the simulated mechanical modes are collective oscillations of several pillars of the mirror region (not shown), which is a consequence of their identical geometry and the slight mechanical coupling through the substrate. Those mechanical modes do not exhibit significant $g_{OM}/2\pi$ values given that the overlap with the optical mode is modest.

Finally, we discuss the role of the spatial distribution of the optical mode on tailoring the RF spectra that we reported experimentally in Figure 4c of the main text. There we show that the relative signal strength associated with each mechanical peak vary with the optical mode used to

excite the cavity and associate that observation to a different electromagnetic field spatial distribution along the 1D-PhC pillar cavity. Figure S6a and Figure S6b report that the spatial distribution of the fundamental and second order optical mode extracted from the FEM simulations are clearly different, showing that the second order mode occupies more volume than the fundamental, even extending to pillars of the mirror region. The values of $g_{OM}/2\pi$ associated with the mechanical modes of the first family are therefore different for each of the optical modes considered. Given that the mechanical frequencies increase with the distance of the oscillating pillars from the centre, the frequency spectrum of $g_{OM}/2\pi$ roughly follows the spatial distribution of the optical field in the cavity region.

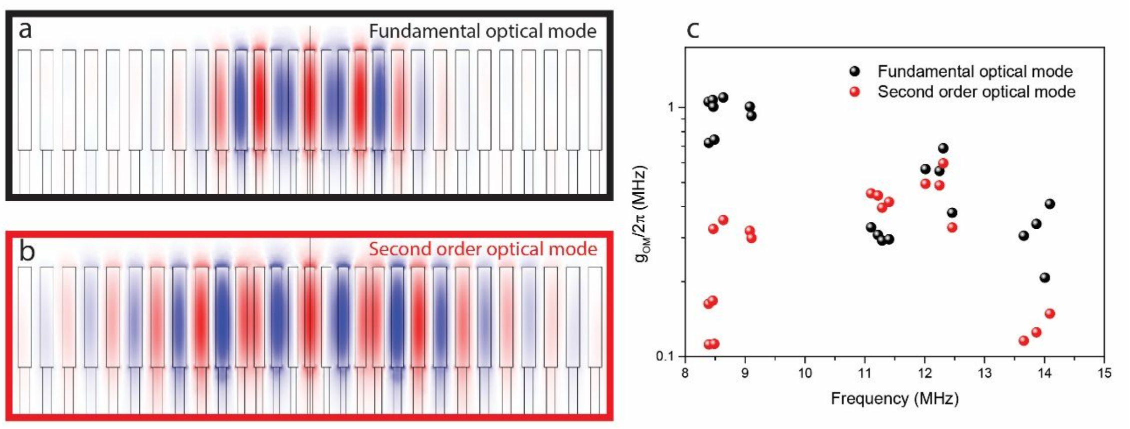

Figure S6. a) and b) Finite-Element-Method simulation of the electric field along the z direction (E_z) of the TM fundamental and second order optical cavity mode (panel a and b, respectively) as seen from the side. c) Vacuum OM coupling ($g_{OM}/2\pi$) calculations for the fundamental and second order optical cavity modes supported by the cavity (black and red dots, respectively). The mechanical modes are the same for both calculations.

S4. Estimation of the responsivity of the mechanical eigenfrequencies to applied force derivatives.

In the main text of the manuscript, Figure 5 demonstrates the potentiality of our geometries for force sensing applications by showing that specific mechanical modes of the 1D-PhC frequency-shift towards larger frequencies when the fiber comes into contact with some of the pillars. The amount of the shift is directly related with the pressure that the fiber applies on the pillars.

To give a quantitative estimation of the response of the mechanical frequencies to force derivatives against deformation we use FEM simulations and apply an elastic boundary condition to the top surface of a pillar (see inset of Figure S6). We have tracked the mechanical frequency value as a function of the force derivative. To get a shift on the order of 10-20 MHz for the second family of modes, which is what measured in Figure 5 of the main text, the force derivative, i.e., increase of the elastic constant of this type of mechanical mode, must be about few N/m.

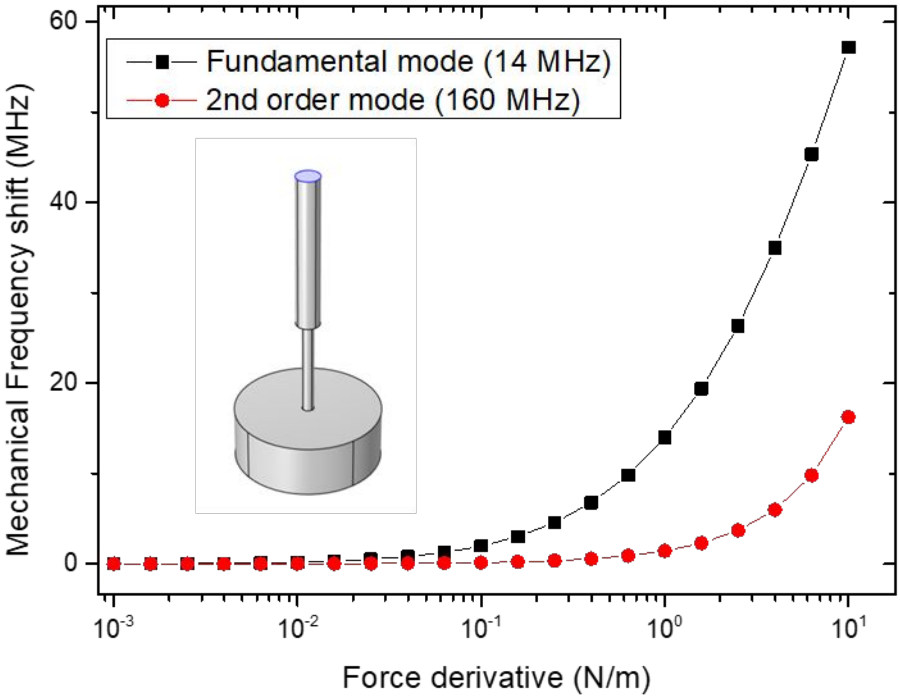

Figure S7. Finite element method simulations of the response of the mechanical eigenfrequency of a nanopillar to force derivatives applied on the top part of the pillar (see inset). The black and red curves correspond to the first and second order mode, respectively.

References:

1. Johnson, S. G. *et al.* Perturbation theory for Maxwell's equations with shifting material boundaries. *Phys Rev E* **65**, 66611 (2002).

2. Chan, J., Safavi-Naeini, A. H., Hill, J. T., Meenehan, S. & Painter, O. Optimized optomechanical crystal cavity with acoustic radiation shield. *Appl Phys Lett* **101**, 81115 (2012).
